# The role of postmastectomy radiation therapy in patients with immediate prosthetic breast reconstruction: A meta-analysis

**DOI:** 10.1097/MD.0000000000009548

**Published:** 2018-02-09

**Authors:** Yun Pu, Tong-Chun Mao, Yi-Ming Zhang, Shao-liang Wang, Dong-Li Fan

**Affiliations:** Department of Plastic Surgery, Xinqiao Hospital, Third Military Medical University, Chongqing, China.

**Keywords:** complications, immediate breast reconstruction, meta-analysis, PMRT, radiation therapy

## Abstract

**Objective:**

The controversy remains as to whether immediate prosthetic breast reconstruction with postmastectomy radiation therapy (PMRT) is associated with acceptable complications and aesthetic outcomes. The aim of this meta-analysis was to provide a pooled analysis of comparative clinical studies that evaluated breast cancer patients who were treated with a mastectomy and an immediate prosthetic breast reconstruction to compare the complications and satisfaction of those who underwent or did not undergo PMRT.

**Methods:**

According to the recommendations of the Cochrane Collaboration, we established a rigorous study protocol. We performed a systematic electronic search of the PubMed and Embase databases to identify articles for inclusion in our meta-analysis. Reconstruction failure, overall complications, capsular contracture, and patient satisfaction were analyzed individually.

**Results:**

Fifteen controlled trials were included, comprising 5314 patients (1069 PMRT vs 4245 non-PMRT). Primary outcomes revealed a statistically significant increase in overall complications [odds ratio (OR) 3.45; 95% confidence interval (95% CI) 2.62–4.54; *P* < .00001], reconstruction failure (OR: 2.59; 95% CI 1.46–4.62; *P* = .001), and capsular contracture (OR: 5.26, 95% CI: 2.73–10.13, *P* < .00001) after receiving PMRT.

**Conclusion:**

Our review found that PMRT for patients who underwent immediate implant-based breast reconstruction led to higher risks of reconstruction failure, overall complications, and capsular contracture. However, it is still the standard adjuvant therapy for mastectomy patients who have opted for immediate implant-based breast reconstruction.

## 1 Introduction

Currently, radiation therapy is the standard of care for breast cancer treatment following lumpectomy.^[1]^ It may be utilized either following lumpectomy or after mastectomy, and postmastectomy radiation therapy (PMRT) in patients with locally advanced breast cancer has been demonstrated to improve both local control and patient survival.^[2]^ As a result, PMRT is routinely used to prevent local recurrence in patients who have close or positive mastectomy margins or to obtain long-term, local-regional control in patients who have suffered a chest wall recurrence.^[3,4]^

As proven in women treated for primary breast cancer, immediate breast reconstruction, performed at the time of mastectomy, is a procedure that offers good clinical, aesthetic, and psychological results and is oncologically safe in terms of local recurrence and long-term survival rates.^[4–6]^ Two major breast reconstruction options are currently available, namely autologous reconstruction and implant-based reconstruction. Implant reconstruction has a major role in immediate breast reconstruction today because some patients do not wish to undergo a major surgical procedure and others are deemed to be unsuitable candidates for autologous reconstruction.

Indeed, the presence of a reconstruction can complicate plans for radiation therapy. Similarly, the effects of radiation therapy on the immediate breast reconstruction has generated significant discussion and controversy. Several studies have demonstrated that PMRT may compromise aesthetic outcomes and increase the complication rates for immediate breast reconstruction, especially in implant breast reconstructions.^[7–9]^ These authors reported that PMRT engenders changes in mastectomy flap perfusion, which may result in infection, tissue necrosis, capsular contracture, implant extrusion, wound dehiscence, and complete reconstructive failures. However, other studies^[10]^ have demonstrated that there is no significant difference in the overall rate of major or minor complications between the PMRT group and non-PMRT group.

Despite these established complications, many patients seem to successfully undergo both PMRT and breast reconstruction when treated with a systematic and carefully considered approach. The objective of this study was to analyze whether the following outcomes were affected by PMRT: reconstruction failure, overall complications, capsular contracture, and the patients’ satisfaction with cosmetic and functional outcomes. Knowledge of these outcomes will allow clinicians to provide women who are candidates for immediate implant breast reconstruction with information about the risks and benefits of PMRT.

## 2 Materials and methods

We performed a systematic electronic search of PubMed, Embase, the Cochrane Library databases, Web of Science, Chinese Biomedical Database, and Chinese Scientific Journals database to identify articles for inclusion in our meta-analysis. And ethical approval was not necessary for this meta-analysis. The search terms “breast reconstruction,” “radiation therapy,” “immediate,” and “mastectomy” and the Medical Subjects Headings (MeSH) terms of “breast reconstruction” (MeSH), “radiation therapy” (MeSH), “immediate” (MeSH), and “mastectomy” (MeSH) were used in combination with the Boolean operators AND or OR. The electronic search was supplemented by a hand-search of published abstracts from the annual meetings of relevant surgical societies. Reference searches were also conducted, where lists of trials selected from the electronic search were scanned to identify further relevant trials.

Abstracts of the citations that were identified by the search were then scrutinized by 2 observers to determine the eligibility of the corresponding study for inclusion in the meta-analysis. Studies were included if they met each of the following criteria: it was a comparative study, and the study sample was separated into groups based on the use or nonuse of PMRT among patients with immediate implant breast reconstructions. Studies without non-PMRT controls were excluded from our study. Our search identified 15 studies that met our criteria in the meta-analysis. The data extracted from each article included the following: the study design, number of subjects, male/female ratio, mean age of subjects, and any preoperative interventions performed.

## 3 Statistical analysis

The data from the eligible trials were entered into a computerized spreadsheet for analysis. The quality of each trial was assessed using the Jadad scoring system. We performed the meta-analysis using RevMan 5.1.9 software (provided by the Cochrane Collaboration, Oxford, UK) for the controlled studies. The relative risk (RR) was calculated with a 95% confidence interval (95% CI). We used χ^2^ to assess statistical heterogeneity and the Higgins *I*^2^ statistic to determine the percentage of total variations across studies due to heterogeneity. If the *I*^2^ statistic was ≤ 50%, then a fixed-effect model was used to pool studies; otherwise, a random-effects model was used.

## 4 Results

### 4.1 Study characteristics

**Figure 1:**
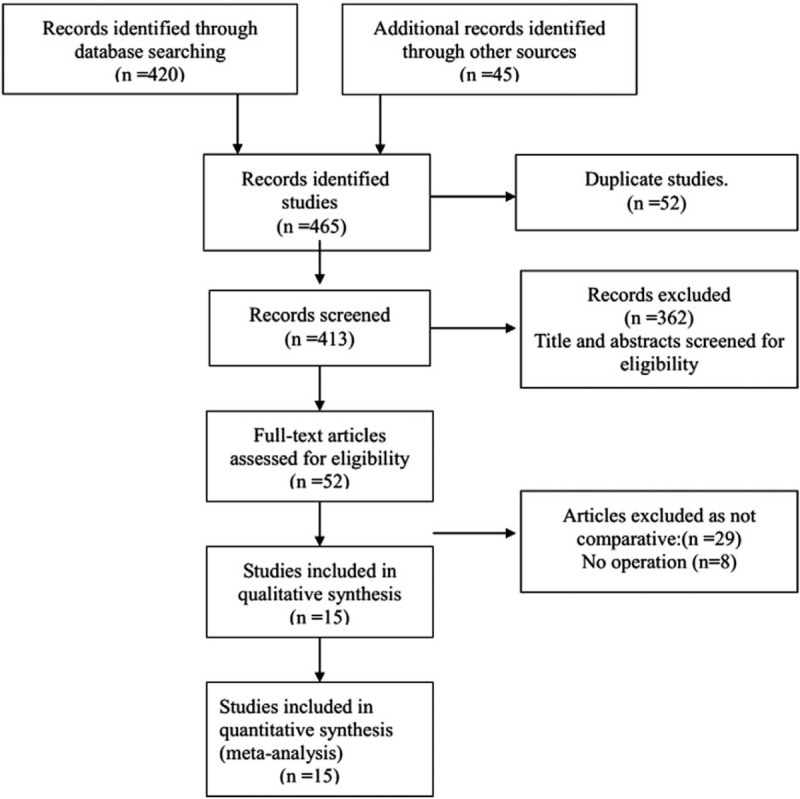
Flow chart of the literature search according to PRISMA.

A total of 15 studies were included.^[11–25]^ All eligible studies were published between 2000 and 2016. Table 1 summarizes the details of each trial, including baseline characteristics, the publication year of the study, the surgical method, and tumor stage for each trial. A PRISMA flowchart (Fig. 1) describes the details of the literature search for this meta-analysis.

**Table 1:**
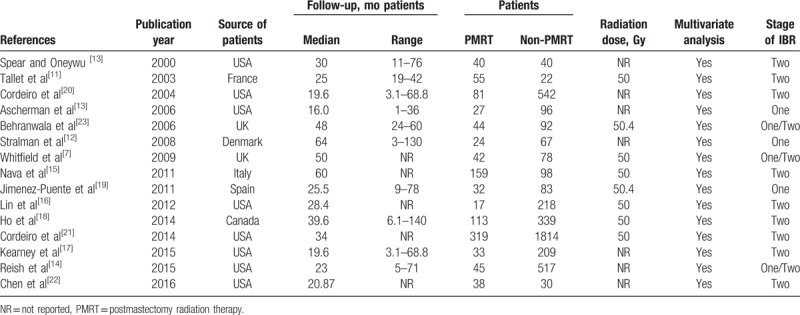
Demographic data.

### 4.2 Reconstruction failure

**Figure 2:**
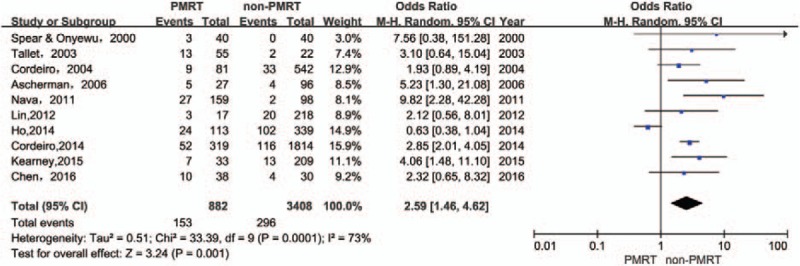
Forest plot for the impact of PMRT on reconstruction failure.

The rates of reconstruction failure of the immediate implant breast reconstructions were available in 10 studies. The use of PMRT increased the rates of reconstruction failure of immediate implant breast reconstruction [odds ratio (OR): 2.59; 95% CI 1.46–4.62; *P* = .001]. Heterogeneity was found to be significant [*I*^2^ = 73%, χ^2^ = 33.39 (df = 9), *P* = 0.001] (Fig. 2).

### 4.3 Overall complications

**Figure 3:**
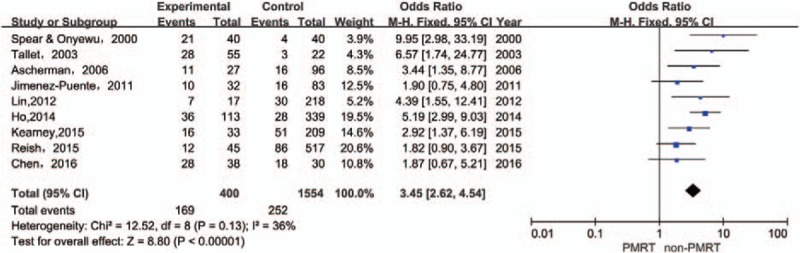
Forest plot for the impact of PMRT on overall complications.

Overall complications were measured in 9 studies, totaling 495 patients with PMRT and 1752 patients without PMRT. It was found that there was a significant difference in the overall complications between those treated with PMRT and without PMRT (OR 3.45; 95% CI 2.62–4.54; *P* < .00001). Statistical heterogeneity was not detected (*I*^2^ = 36%, χ^2^ = 12.52, df = 8, *P* = .13) (Fig. 3).

### 4.4 Capsular contracture

**Figure 4:**
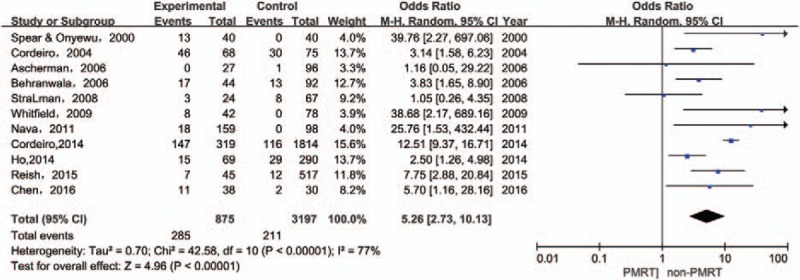
Forest plot for the impact of PMRT on capsular contracture.

Eleven studies reported the incidence of capsular contracture after immediate implant breast reconstruction. The analysis found a significant positive correlation between receiving PMRT and the formation of capsular contractures (OR: 5.26, 95% CI: 2.73–10.13, *P* < .00001). Heterogeneity was found to be significant [*I*^2^ = 77%, χ^2^ = 42.58 (df = 10), *P* < .00001] (Fig. 4).

### 4.5 Patient satisfaction

**Figure 5:**
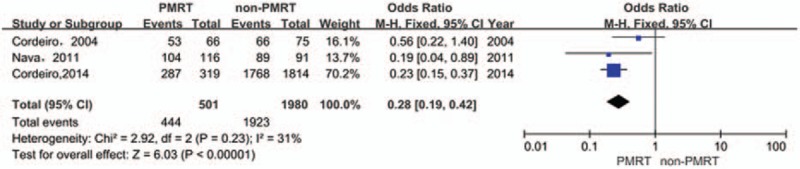
Forest plot for the impact of PMRT on patient satisfaction.

The patient satisfaction outcomes were available in 3 studies. There were significant differences in patient satisfaction between the PMRT and the non-PMRT groups (OR: 0.28, 95% CI: 0.19–0.42; *P* < .00001). There was no evidence of statistical heterogeneity [*I*^2^ = 31%, χ^2^ = 2.92, (df = 2), *P* = .23] (Fig. 5).

### 4.6 Publication bias

**Figure 6:**
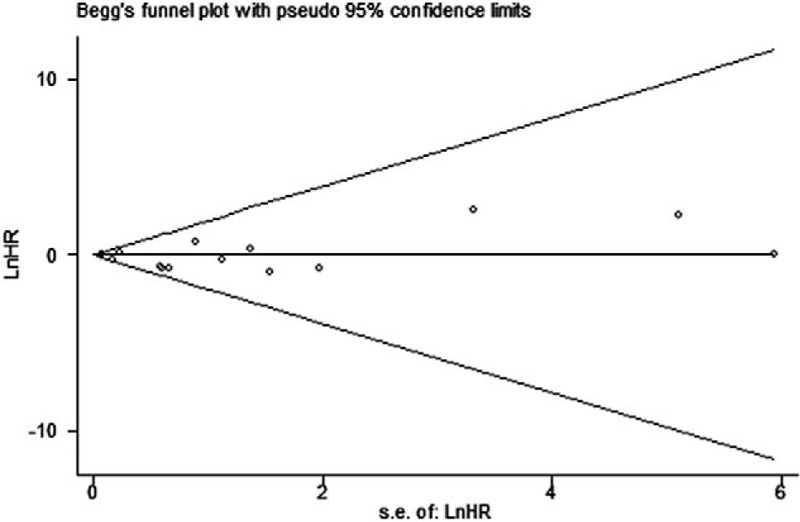
Funnel plot for publication bias.

Funnel plots (for log OR) were drawn for each outcome. The graphical checks showed no asymmetry of the funnel plots. Assessment with the Egger method revealed no evidence of publication bias for any outcomes (*P* = .402, Fig. 6).

## 5 Discussion

During the past decade, immediate implant-based breast reconstruction has become a widely accepted method for breast reconstruction after mastectomy. It has an acceptable rate of postoperative complications and is considered oncologically safe with a local recurrence rate ranging from 2% to 10%. It is well established that PMRT is increasingly used and improves survival for patients with breast cancer. As the indications for PMRT increase, there will be an increase in the number of patients opting for breast reconstruction, who will require radiotherapy as part of their treatment. However, a concern could be raised regarding the possible negative effects of radiotherapy on long-term outcomes after breast reconstruction. In general, the impact of PMRT on implant-based breast reconstruction has been documented in multiple studies over many years, but it continues to be a topic of discussion. Specifically, concerns still exist regarding complication rates, reconstruction failure, capsular contracture, and patient satisfaction, and there is debate regarding the results obtained by delivering PMRT to the permanent implant.

Several studies found that patients with immediate implant-based breast reconstructions who received PMRT had worse outcomes than patients who did not receive PMRT. Multiple complications have been reported in these studies for patients who received PMRT, including infection, tissue necrosis, capsular contracture, implant extrusion, wound dehiscence, and complete reconstruction failures. One of the main complications has been the formation of capsular contractures, leading to poor cosmetic outcome, an increased reoperation rate, and a loss of the breast reconstruction. A study by Spear and Onyewu^[13]^ evaluated patients with immediate implant-based reconstructions and compared PMRT with non-PMRT patients; it was the first study to evaluate the impact of PMRT on immediate implant-based reconstructions. Over their 10-year experience, they reported total complication rates of 52.5% (21 of 40) and 10% (4 of 40) for the PMRT and non-PMRT groups, respectively. In particular, the incidence of capsular contracture was 32.5% for patients who underwent PMRT compared with 0% for those who did not receive PMRT. A limitation of that study is the lack of patient satisfaction outcome data. Similarly, Cordeiro et al^[20]^ conducted a larger series (n = 623) that compared patients who received PMRT (n = 81) with a control group of 542 non-PMRT patients. The incidence of capsular contracture was also significantly higher in the PMRT group. However, there was little difference in patient satisfaction between groups. Although many previous studies have shown that the use of PMRT results in a significantly higher complication rate in patients who underwent immediate implant-based breast reconstruction, 2 recent series demonstrated acceptable results in uniformly treated samples.^[12,18]^ Stralman et al^[12]^ performed a retrospective study (n = 91) that compared patients who received PMRT (n = 24) with a control group of 67 non-PMRT patients. Among the patients who received PMRT, 3 (13%) developed capsular contracture, and among the patients who did not receive PMRT, 8 (12%) developed capsular contracture. The authors found no significant increase in the occurrence of capsular contracture in their study of patients who received PMRT.

As the present review details, the vast majority of studies have been small, retrospective case series that lack the statistical power to make a clear statement regarding the utility of PMRT. A meta-analysis, such as that performed in this study, is a potentially useful tool because it pools data into a very powerful study. In this meta-analysis, we selected all well-controlled cohort studies that evaluated breast cancer patients who were treated with a mastectomy and an immediate prosthetic breast reconstruction and that compared the complications and satisfaction of those who underwent and did not undergo PMRT. This is the first meta-analysis to focus on the risks of PMRT with respect to each type of complication encountered with immediate implant-based breast reconstruction, and it provides a clear trend based on 15 well-controlled cohort studies that were published in the past 15 years, comprised of more than 8200 prosthetic reconstruction cases.

Not surprisingly, we found a significant trend toward higher rates of total complications, capsular contracture, and reconstruction failure in patients who received PMRT as we have previously observed. Furthermore, we also observed a higher rate of grade III/IV capsular contractures that require capsulotomy among patients with reconstructions who received PMRT. We also demonstrated an increased rate of severe capsular contractures and worse cosmetic and satisfaction results among patients who received PMRT.

Although complication rates were higher in patients who received PMRT, the reconstruction failure rate was acceptably low (11.1%), and rates of capsular contracture (30.7%) were acceptable in light of the high patient satisfaction. On the basis of the evidence from this meta-analysis, we think that patients who are motivated enough should certainly undergo immediate breast reconstruction, particularly if the approach that we have outlined is followed carefully. Patients with advanced breast cancers often do not have the opportunity to return for delayed reconstruction because of disease progression. We propose that undergoing both immediate implant-based breast reconstruction and PMRT is still quite acceptable.

This meta-analysis has several potential limitations that should be taken into account. First, a limitation of the current study is the quality of the supporting literature. No randomized trials address the efficacy of PMRT. All the published data were obtained from retrospective cohort studies. Second, only English language articles were considered in our analysis. If the search had been extended to include literature published in other languages, then it is possible that additional relevant trials may have been identified. In addition, many of the aforementioned studies are limited by a small number of patients; heterogeneous patient populations; and variations in the timing, dosage, and duration of irradiation.

## 6 Conclusion

Our study found that PMRT for patients who underwent immediate implant-based breast reconstruction resulted in higher risks of total complications, capsular contracture, and reconstruction failure. However, PMRT is still the standard adjuvant therapy for mastectomy patients who have opted for immediate implant-based breast reconstruction. Thus, it is important that patients are well prepared and preoperatively counseled for the possibility of these complications. A longer follow-up is warranted to assess recurrent cancer, revision surgery, and reconstruction failure.
